# Kv7(KCNQ)-K^+^-Channels Influence Total Peripheral Resistance in Female but Not Male Rats, and Hamper Catecholamine Release in Hypertensive Rats of Both Sexes

**DOI:** 10.3389/fphys.2018.00117

**Published:** 2018-02-20

**Authors:** Torill Berg

**Affiliations:** Division of Physiology, Department of Molecular Medicine, Institute for Basic Medical Sciences, University of Oslo, Oslo, Norway

**Keywords:** Kv7(KCNQ)-K^+^-channels, gender, female, catecholamines, total peripheral vascular resistance, spontaneously hypertensive rats, hypertension

## Abstract

K^+^-channels of the Kv7/KCNQ-family hyperpolarize and stabilize excitable cells such as autonomic neurons and vascular smooth muscle cells (VSMC). Kv7 may therefore play a role in blood pressure (BP) homeostasis, and prevent a high total peripheral vascular resistance (TPR), a hallmark of hypertensive disease. The present study analyzed if Kv7 channels influence catecholamine release and TPR in normotensive (WKY) and spontaneously hypertensive rats (SHR), and if they may contribute to the antihypertensive protection seen in young, female SHR. Tyramine-stimulated norepinephrine release evokes an adrenergic cardiovascular response, and also allows modulation of release to be reflected in the overflow to plasma. The experiment itself activated some secretion of epinephrine. The results show: (1) XE-991 (Kv7.1-7.4-inhibitor), but not chromanol 293B (Kv7.1-inhibitor), increased tyramine-stimulated norepinephrine overflow and epinephrine secretion in both sexes in SHR, but not WKY. (2) Surprisingly, the Kv7-openers retigabine (Kv7.2-7.5) and ICA-27243 (Kv7.2-7.3-preferring) increased catecholamine release in female SHR. (3) The rise in TPR following tyramine-stimulated norepinephrine release was increased by XE-991 but not chromanol in the female WKY only. (4) Retigabine and ICA-27243 reduced the TPR-response to tyramine in the female SHR only. These results suggested: (1) Up-regulation of Kv7.2-7.3 function in sympathetic neurons and chromaffin cells hampered catecholamine release in SHR of both sexes. (2) The increase catecholamine release observed after channel openers in the female SHR may possibly involve reduced transmission in cholinergic neurons which hamper catecholamine release. These two mechanisms may serve to counter-act the hyperadrenergic state in SHR. (3) Kv7.4, most likely in the vasculature, opposed the tension-response to norepinephrine in the female WKY. (4) Vascular Kv7.4-7.5 could be stimulated and then opposed norepinephrine-induced vasoconstriction in the female SHR. (5) Vascular Kv7 channels did not counter-act norepinephrine induced vasoconstriction in male rats, possibly due to different Kv7 channel regulation. Kv7 channels may represent a novel target for antihypertensive therapy.

## Introduction

Sympathetic nerve hyperactivity plays an important role in the pathology of hypertension, and can be detected years before the clinical occurrence of a high blood pressure (BP) (Julius, 1992). This is true also in women (Hall et al., 2015), in spite of that premenopausal women and also young, female, spontaneously hypertensive rats (SHR) carry protective mechanism(s) which delay(s) the development of the hypertensive disease (Lerner and Kannel, 1986; Maris et al., 2005; Berg, 2016a). Increased sympathetic nerve discharge results in an elevated total peripheral vascular resistance (TPR), a hallmark of essential hypertension. It also increases heart rate (HR), and a high resting HR is the most reliable predictor of cardiovascular morbidity in man (Palatini, 1999).

**Figure 1:**
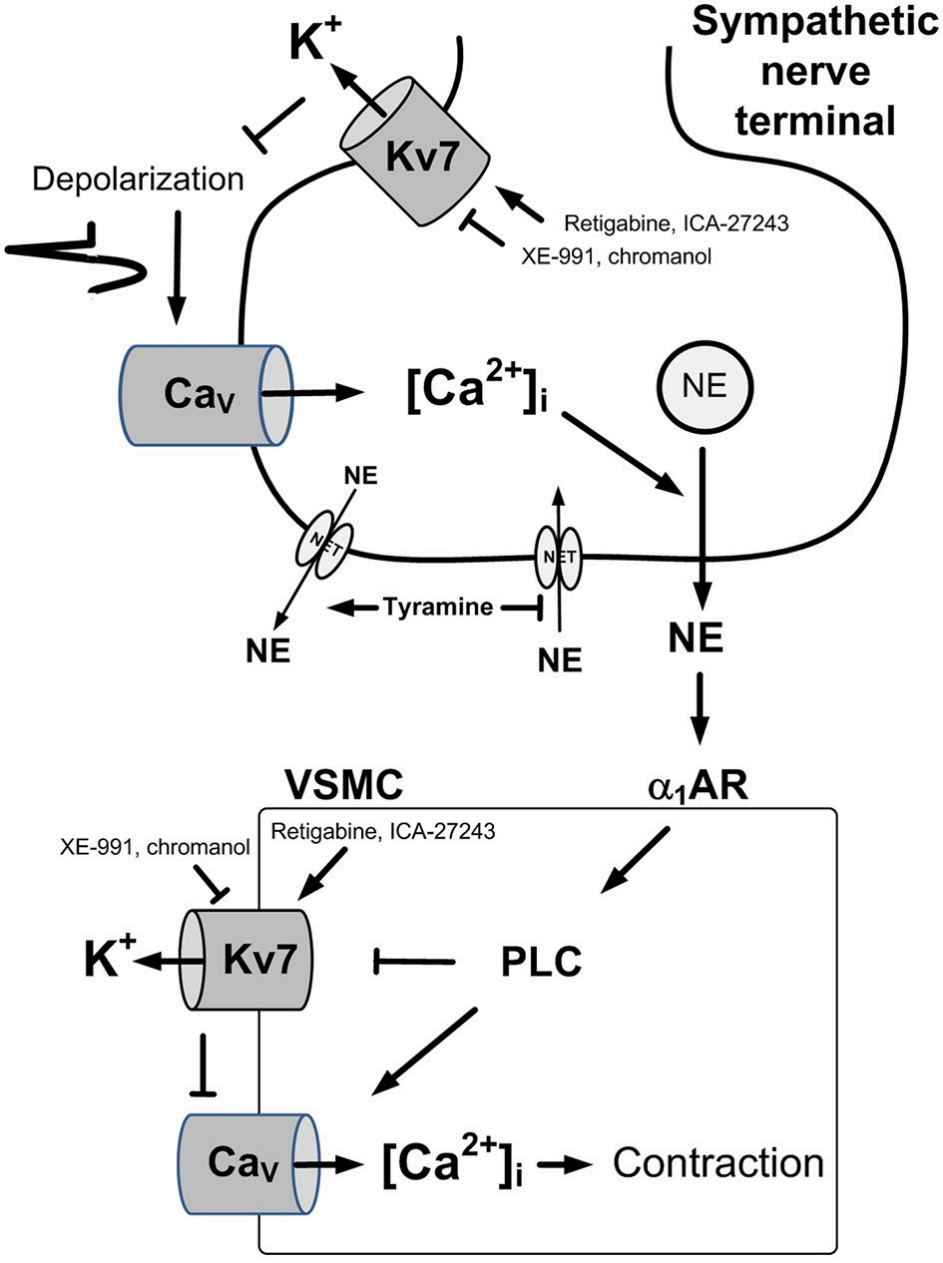
Control of norepinephrine release and VSMC tension. A rise in [Ca^2+^]_i_ stimulates vesicular release of norepinephrine in sympathetic nerve terminals **(Upper)** and precipitates VSMC contraction through activation of myosin light chain kinase **(Lower)**. A rise in [Ca^2+^]_i_ occurs in the nerves in response to depolarization which activates the entry of Ca^2+^ through L-type voltage-sensitive Ca^2+^ (Ca_v_). In VSMC, a rise in [Ca^2+^]_i_ occurs through activation of the PLC pathway, stimulated by for instance by the α_1_AR. The PLC pathway allows the release of Ca^2+^ from intracellular stores (not indicated), or the entry of Ca^2+^ through Ca_v_, either directly, or through depolarization following inhibition of Kv7 channels. Hyperpolarizing Kv7 channels will therefore stabilize both cell types and prevent transmitter release and VSMC contraction. Pointed arrows—stimulation. Blunted arrows—inhibition. NE, norepinephrine; NET, norepinephrine re-uptake transporter.

Transmitter release from sympathetic nerve terminals and contraction of vascular smooth muscle cells (VSMC) are both activated by a rise in the intracellular concentration of Ca^2+^ ([Ca^2+^]_i_). In nerve terminals, this occurs during depolarization, which opens voltage-sensitive Ca^2+^ channels (Ca_V_), thus allowing the entry of Ca^2+^ (Figure 1). In VSMC, a rise in [Ca^2+^]_i_ may occur in response to activation of the phospholipase C (PLC) pathway, activated, among others, by α_1_-adrenoceptors (AR) (Figure 1). The PLC pathway may open Ca_V_ directly, or, through inhibition of K^+^ channels, induce depolarization, and in that manner cause Ca_V_ to open. Outward directed K^+^ channels promote hyperpolarization, and therefore oppose depolarization, and thus hamper neuronal and VSMC excitability. Voltage-sensitive K^+^ channels (Kv) of the Kv7 (KCNQ) family, i.e., Kv7.2-7.3 (KNCQ2-KNCQ3) (Wang et al., 1998), also called M-channels, are known to suppress neuronal excitability and transmitter release throughout the nervous system (Brown and Passmore, 2009). Members of the Kv7-family, i.e., Kv7.1, 7.4, and 7.5, have also been demonstrated in rat arterial VSMC, where they promote vasodilation (Mackie et al., 2008). It may therefore be postulated that a reduction in M-currents in sympathetic nerves and/or VSMC may contribute to the development of hypertension.

Little has been done so far to explore this hypothesis *in vivo*. However, the Kv7.1–7.4 inhibitor XE-991 surprisingly increased norepinephrine and epinephrine release in SHR, activated by depolarization induced by a non-Kv7 Kv blocker (3,4-diaminopyridine) (Berg, 2016b). This observation suggested in fact an up-regulated role of Kv7 channels in hampering the release of catecholamines in SHR. Studies on pre-constricted, isolated arteries have demonstrated a reduced Kv7.4-mediated vasodilation in SHR compared to that in normotensive rats (Jepps et al., 2011; Chadha et al., 2012). However, a similar strain-related difference in the TPR-response to Kv7 inhibitors/openers was not detected *in vivo* in unstimulated male rats (Berg, 2016b). Since Kv7 channels are more likely to be open during depolarization, this difference may result from the low sympathetic tone in pentobarbital-anesthetized rats. The role of Kv7 channels in TPR and BP control may therefore be studied preferably during norepinephrine-induced VSMC constriction.

Activation of norepinephrine release can be achieved *in vivo* by tyramine, which stimulates the release of norepinephrine by reversing the transport through the norepinephrine re-uptake transporter (NET) (Berg, 2014b) (Figure 1). Since NET then is engaged in release, synaptic re-uptake is prevented, and presynaptic modulation of concomitant vesicle norepinephrine release is reflected as differences in norepinephrine overflow to plasma (Berg et al., 2012; Berg and Jensen, 2013). The experiment itself induced some secretion of epinephrine, also subjected to modulation (Berg et al., 2012). The use of tyramine will therefore provoke an adrenergic cardiovascular response and at the same time allow demonstration of modulation of catecholamine release. The role of Kv7 channels in these functions may be visualized by pre-treatment with Kv7 inhibitors or openers.

The purpose of the present study was therefore, through a pharmacological approach, to investigate *in vivo* the possible impact of Kv7 channels on the cardiovascular response and catecholamine release to tyramine-stimulated norepinephrine release in SHR compared to that in the normotensive controls (WKY). Since increased levels of vascular Kv7.4 and their positively regulating protein KNCE4 have been demonstrated *in vitro* in female mice (Abbott and Jepps, 2016), VSMC Kv7 may play a role in the low TPR and BP seen in young women and female SHR (Lerner and Kannel, 1986; Maris et al., 2005; Berg, 2016a). The experiments were therefore carried out in both female and male rats.

## Materials and methods

### Preparation of animals

All experiments were conducted in accordance with the European Directive 2010/63/EU. The protocol was approved by The Norwegian Animal Research Authority (NARA). SHR (Okamoto, SHR/NHsd strain) (41 females, 162 ± 2 g, 12.7 ± 0.1 months and 47 males, 246 ± 7 g, 12.7 ± 0.1 months) and WKY (Wistar Kyoto) (40 females, 166 ± 1 g, 12.4 ± 0.1 months and 35 males, 252 ± 3 g, 12.7 ± 0.1 months) were included in the study. The rats were originally obtained from formally legacy Harlan, now Envigo RMS, Bicester, Oxfordshire, UK, and bred in-house. The rats were kept on a 12/12 h day/night cycle and fed with Teklad Global 18% Protein Rodent Diet (Teklad Diets, Madison, WI, USA) containing 0.2% Na^+^. The rats were anesthetized with pentobarbital (65–75 mg/kg IP). Systolic (SBP) and diastolic (DBP) BP and HR were recorded through a catheter in the femoral artery. When starting BP and HR had been recorded, the rats were connected to a positive-pressure ventilator. Thoracotomy was performed through the third intercostal space, and a 2SB perivascular flow probe, connected to a T206 Ultrasonic Transit-Time Flowmeter (Transonic Systems Inc., Ithaca, NY, USA), was placed on the ascending aorta to measure cardiac output (CO, i.e., without cardiac flow) and from now on also HR. MBP (SBP-DBP/3+DBP) and TPR (TPR = MBP/CO) were calculated. Body temperature was maintained at 37–38°C by external heating, monitored by a thermo sensor inserted inguinally 1–2 cm into the abdominal cavity. The arterial catheter was subsequently flushed with 0.15 ml buffered saline (PBS; 0.01 M Na-phosphate, pH 7.4, 0.14 M NaCl) containing 500 I.U./ml heparin, and PBS injected to stabilize BP. A stabilization period of 10 min was allowed before drug injections were started.

### Experimental design

Reverse transport of norepinephrine through NET was activated by tyramine, which does not cross the blood-brain barrier (Berg, 2014b) (Figure 1). Since NET then is engaged in release, synaptic re-uptake is prevented, and presynaptic modulation of concomitant vesicular release is reflected as differences in norepinephrine overflow to plasma (Berg et al., 2012; Berg and Jensen, 2013). Kv7 channels, through their hyperpolarizing action, may influence this modulation. Tyramine-stimulated norepinephrine overflow was not different after acute AdrX (Berg, 2014a), indicating that the plasma norepinephrine concentration mainly originated from sympathetic nerves. The amount of norepinephrine released into the synapse is likely to be more than that needed to elicit a maximum cardiovascular response. Drug-induced differences in the TPR-response to tyramine will therefore reflect the direct effect of the drug on VSMC tension control. The cardiovascular response to pre-treatment and tyramine was not influenced by baroreflex activation, since the use of pentobarbital anesthesia eliminated such reflexes, indicated by the absence of a HR-response to large, bradykinin-induced, acute reductions, or phenylephrine-induced increases in MBP (Bjørnstad-Ostensen and Berg, 1994; Berg et al., 2012). This was further confirmed by the fact that ganglion blockade did not alter the cardiovascular response to tyramine and also had only little effect on the tyramine-induced norepinephrine over-flow (Berg, 2005). The experiment itself induced some secretion of epinephrine, also subjected to modulation (Berg et al., 2012).

### Experimental protocols

Control rats were pre-treated with PBS, and 10 min later infused with tyramine for 15 min (1.26 μmol/kg/min) to stimulate the release of norepinephrine (Berg, 2005; Berg and Jensen, 2013). In the experimental groups, the PBS-sham injection was substituted either by Kv7 inhibitor to detect a possible Kv7 channel influence on the response to tyramine, or by Kv7 opener to indicate the presence of Kv7 channels and their ability to respond to channel opener. Kv7 channels are present in many organs and have many different functions, but organ- or subtype-selective Kv7 inhibitors or openers are not available. The neuronal Kv7 channels are predominantly of the Kv7.2-/7.3 subtype, and VSMC Kv7 channels of the Kv7.1, 7.4 and 7.5 subtypes. In this early attempt to analyse the role of Kv7 channels in blood pressure control and hypertension *in vivo*, pre-treatment included the Kv7.1–7.4-inhibitor XE-991 (2.2 μmol/kg, −10 min), the Kv7.1 (K_s_) inhibitor chromanol 293B (3 μmol/kg, −10 min), the Kv7.2-7.5 opener retigabine (4.3 μmol/kg, −25 min), or the Kv7.2-7.3-preferring opener ICA-27243 (11.2 μmol/kg, −10 min) (Wickenden et al., 2000; Yang et al., 2004; Yeung et al., 2007; Qi et al., 2011; Berg, 2016b). The effect of Kv7 inhibitor/opener on cardiovascular baselines was recorded at the time of maximum change in TPR in the period between drug injection and tyramine. The cardiovascular response to subsequent tyramine-stimulated norepinephrine release was recorded throughout the tyramine-infusion period. The impact on the plasma catecholamine concentrations was measured in blood sampled at the end of the tyramine observation-period.

### Measurement of plasma catecholamine concentrations

Blood was collected by free flow from the arterial catheter at the end of the tyramine observation-period without terminating the tyramine-infusion. Plasma was stored at −80°C until the catecholamine concentrations were determined using 400 μl plasma and the “5000 Reagent kit for HPLC analysis of Catecholamines in plasma” (Chromsystems GmbH, Munich, Germany), as described by the manufacturer (Berg, 2014b).

### Drugs

Pentobarbital was obtained from The Norwegian National Hospital, Oslo, Norway; XE-991 dihydrochloride [10,10-bis(4-pyridinylmethyl)-9(10H)-anthracenone] and retigabine [ethyl-(2-amino-4-(4-fluorobenzylamino)-phenyl)carbamate dihydrochloride] from MedChem Express, Princeton, NJ, USA, and ICA-27243 [N-(6-Chloro-pyridin-3-yl)-3,4-difluoro-benzamide] from Alomone Labs, Jerusalem, Israel. The remaining drugs were from Sigma Chemical Co., St. Louis, MO, USA.

### Statistical analyses

All data are presented as mean values ± s.e.m. Each group comprised 6–12 rats. The cardiovascular data recorded throughout the experiments were averaged every minute, except for starting BP and HR, and the initial response to PBS, XE-991, chromanol and retigabine, where data were averaged every 7th heart-beat. The HR-, CO-, and MBP-response to channel drugs was recorded at the same time as the highest/lowest TPR-response was detected. The cardiovascular response to pre-treatment, baselines prior to tyramine, and the plasma catecholamine concentrations were evaluated over-all by one-way ANOVA, including all groups, and then for each set of groups within each sex or strain. When the presence of group differences was indicated, these were located by two-tailed, two-sample Student's *t*-tests for parametric data, and by Kruskal–Wallis tests for non-parametric data. Since TPR is determined by the vessel radius in the fourth power, changes in TPR were expressed in percent of before values. The tyramine-response curves were analyzed by Repeated Measures Analyses of Variance and Covariance, first as overall tests including all groups (*P* ≤ 0.05) or the four sets of groups separately (i.e., female and male WKY and SHR, *P* ≤ 0.0125), then, within each set of groups, i.e., between the control group and the experimental groups (*P* < 0.0125) and for each group separately (*P* ≤ 0.01). Significant responses compared to baselines (one-sample Student's *t*-tests) and group differences (two-tailed, two-sample Student's *t*- or Kruskall–Wallis tests) were subsequently located at specific times, i.e., during the initial TPR-peak-response (at 4 min for TPR and for MBP) and at the end of the experiment (at 15 min for TPR, HR, CO, and MBP). For each step of analyses, testing proceeded only when the presence of significant responses, differences and/or interactions was indicated. The *P*-value was for all tests and each step adjusted according to Bonferroni, except for the plasma catecholamine concentrations, where *P* ≤ 0.05 was considered significant.

## Results

### The role of Kv7 channels in stimulated catecholamine-release (Table 1)

The tyramine-induced norepinephrine overflow was greater in SHR than in WKY in both sexes (*P* ≤ 0.002), and higher in the females than in the males in both strains (*P* = 0.014). The plasma epinephrine concentration in the male WKY was lower than in the female WKY (*P* = 0.021) and also less than in the male SHR (*P* = 0.007). The Kv7.1-7.4 inhibitor XE-991 increased the tyramine-induced norepinephrine overflow and the secretion of epinephrine in both sexes in SHR (*P* ≤ 0.039), but had no effect in either sex in WKY (*P* = NS). The Kv7.1 inhibitor chromanol induced a minor reduction in the plasma catecholamine levels in the female WKY but otherwise had no effect. Surprisingly, an increase in the plasma catecholamine concentrations was detected also after pre-treatment with the Kv7.2-7.5 opener retigabine, but only in the female SHR (*P* ≤ 0.032). The same pattern was seen after pre-treatment with the Kv7.2-7.3-preferring opener ICA-27243 (*P* ≤ 0.039).

**Table 1:** The effect of Kv7 inhibitors/openers on the plasma catecholamine concentrations after tyramine-stimulated norepinephrine release.

**Groups**	**WKY**		**SHR**	
	**Norepinephrine (nM)**	**Epinephrine (nM)**	**Norepinephrin (nM)**	**Epinephrin (nM)**
**FEMALE RATS**				
PBS+tyramine (control)	22.9 ± 1.0	3.9 ± 1.2	32.5 ± 1.3^*^	4.5 ± 1.2
XE-991 (Kv7.1-7.4 inhibitor)+tyramine	21.8 ± 2.4	3.4 ± 1.3	43.7 ± 4.8^‡^	15.5 ± 4.3^‡^
Chromanol (Kv7.1 inhibitor)+tyramine	20.1 ± 0.8^‡^	1.2 ± 0.2^‡^	33.9 ± 0.9	6.0 ± 1.1
Retigabine (Kv7.2-7.5 opener)+tyramine	23.8 ± 2.0	6.4 ± 2.7	45.9 ± 5.6^‡^	22.4 ± 7.5^‡^
ICA-27243 (Kv7.2-7.3 opener)+tyramine	21.8 ± 2.0	1.7 ± 0.2	39.5 ± 1.1^‡^	8.3 ± 1.8^‡^
**MALE RATS**				
PBS+tyramine	17.5 ± 1.7^†^	1.2 ± 0.2^†^	25.0 ± 1.2^*^^†^	6.7 ± 1.6^*^
XE-991+tyramine	22.8 ± 2.1	1.0 ± 0.3	41.7 ± 6.3^‡^	24.1 ± 6.6^‡^
Chromanol+tyramine	21.5 ± 1.0	0.9 ± 0.6	21.5 ± 1.6	9.4 ± 3.6
Retigabine+tyramine	19.2 ± 1.6	1.1 ± 0.3	32.8 ± 4.6	22.2 ± 8.3
ICA-27243+tyramine	19.7 ± 0.9	1.5 ± 0.6	28.1 ± 1.5	7.5 ± 1.3

After one-way ANOVA over-all tests had indicated the presence of group differences in the plasma catecholamine concentrations (P < 0.001), these were detected by two-sample Student's t-tests (parametric data) or Kruskal–Wallis tests (non-parametric data) as indicated. Comparisons were made between SHR and WKY control groups within each sex (^*^after SHR values), between the female and male control groups within each strain (†after male values), between corresponding control and experimental groups (^‡^).

*†‡ *P ≤ 0.05*.

### Cardiovascular baselines

MBP prior to connecting the rats to the ventilator was 80 ± 4 and 102 ± 12 mm Hg in the female WKY and SHR control groups, respectively, and 90 ± 5 and 150 ± 8 mm Hg, in the male WKY and SHR control groups (*P* < 0.001 for a strain-related difference in the males, and *P* = 0.001 for a sex-dependent difference in SHR). HR before the rats were connected to the ventilator was 307 ± 12, 375 ± 9, 329 ± 7, and 378 ± 8 bpm in the four groups, respectively (*P* ≤ 0.001 for a strain related difference in both sexes, *P* = NS for sex-dependent differences). Positive-pressure ventilation reduces venous return to the right heart, and, hence, lowers CO and BP, particularly in SHR. MBP prior to pre-treatment was therefore 65 ± 3, 63 ± 7, 63 ± 4, and 94 ± 6 mm Hg in the female WKY and SHR and male WKY and SHR control groups, respectively (*P* ≤ 0.004 for a sex-related difference in SHR and a strain-related difference in the males). At this time HR = 360 ± 14, 383 ± 9, 363 ± 10, and 407 ± 7 bpm, respectively (*P* = 0.002 for a strain-related difference in the male SHR), CO = 21 ± 2, 17 ± 1, 29 ± 3, and 18 ± 1 ml/min (*P* = 0.003 for a strain-related difference in the male SHR), and TPR = 3.2 ± 0.2, 3.9 ± 0.5, 2.3 ± 0.2, and 5.2 ± 0.4 mm Hg/ml/min (*P* ≤ 0.005 for a sex-related difference in WKY and a strain-related difference in the males).

### The influence of Kv7 on cardiovascular baselines

**Figure 2:**
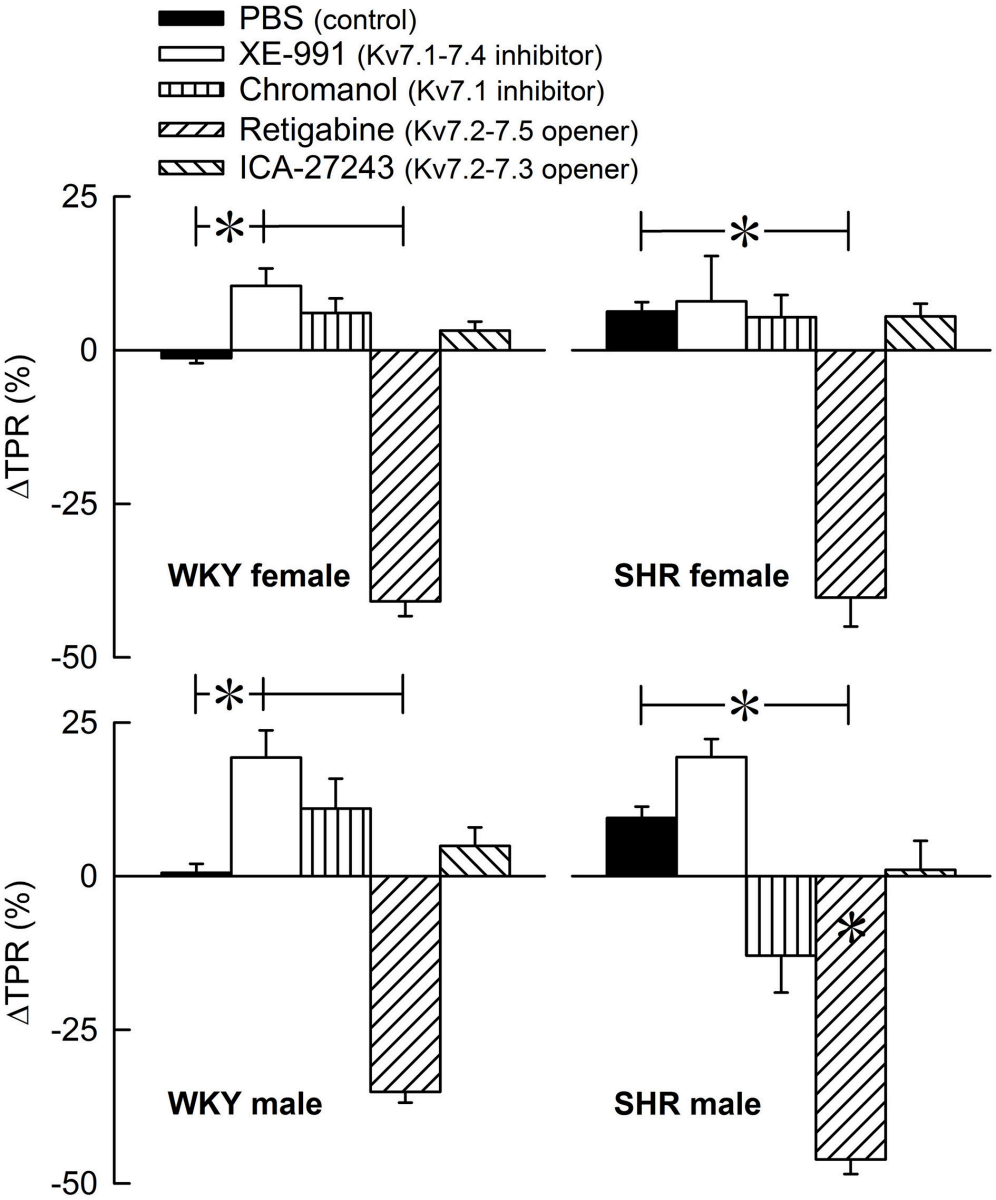
The acute TPR-response to Kv7 inhibitors/openers. The rats were injected with vehicle (PBS) in the controls, and XE-991, chromanol, retigabine, or ICA-27243 in the experimental groups, as indicated by the symbol legends. ΔTPR was recorded at the time of maximum change from baseline. Comparisons were made between corresponding PBS-control and experimental groups, and for corresponding experimental groups across sex (none detected) or strain (^*^within column). Analyses were done by one-way ANOVA, followed by Student's two-sample *t*-tests or Kruskal–Wallis tests. ^*^P ≤ 0.0125.

**Figure 3:**
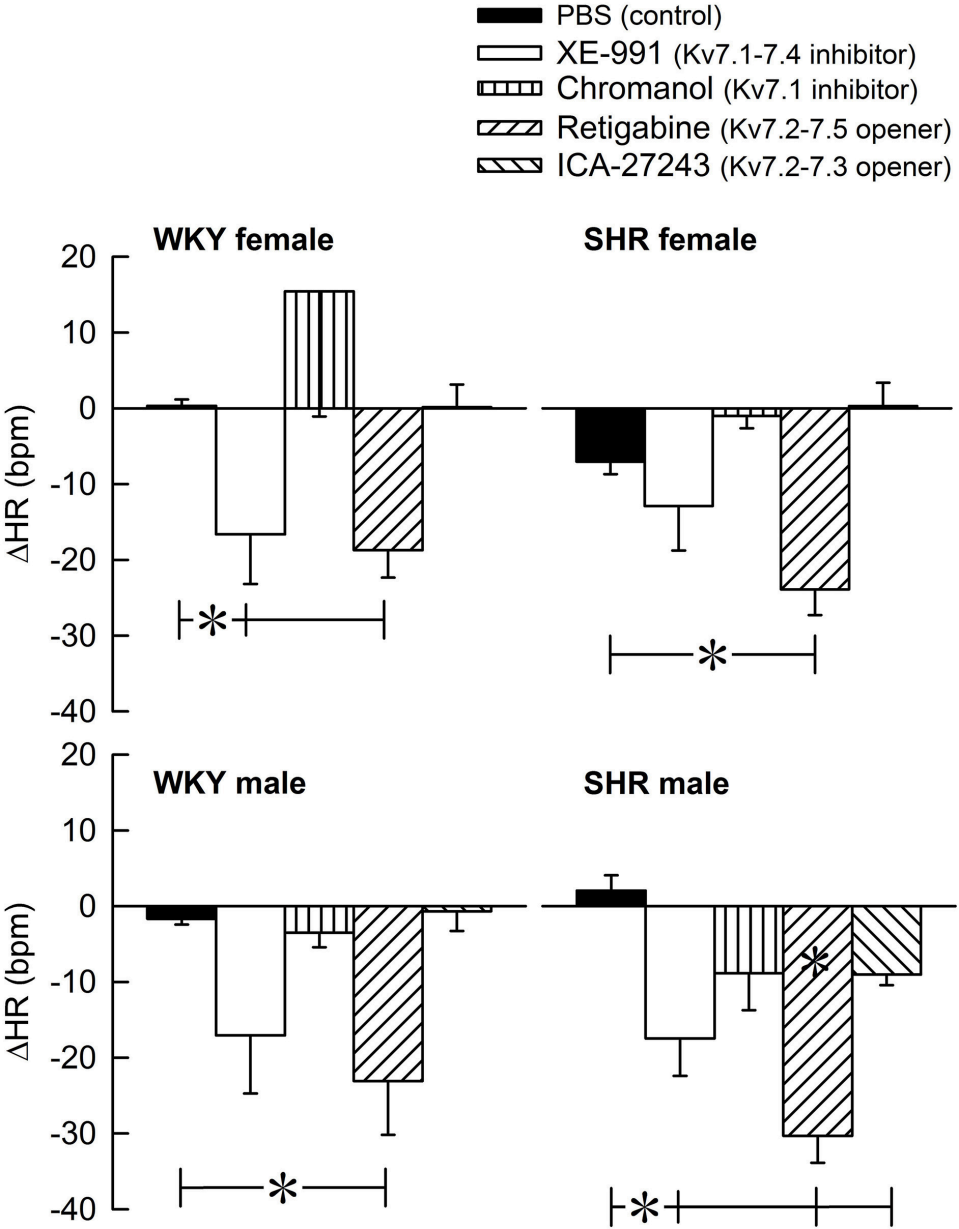
The acute HR-response to Kv7 inhibitors/openers. The rats were pre-treated with PBS (controls) or Kv7 inhibitor or opener, as indicated by the symbol legends. The change in HR compared to baseline was recorded at the time of the maximum change in TPR (Figure 2). Comparisons were made between corresponding PBS-control and experimental groups, and for corresponding experimental groups across sex (none detected) or strain (^*^within column). Analyses were done by one-way ANOVA, followed by Student's two-sample *t*-tests or Kruskal–Wallis test. ^*^P ≤ 0.0125.

**Figure 4:**
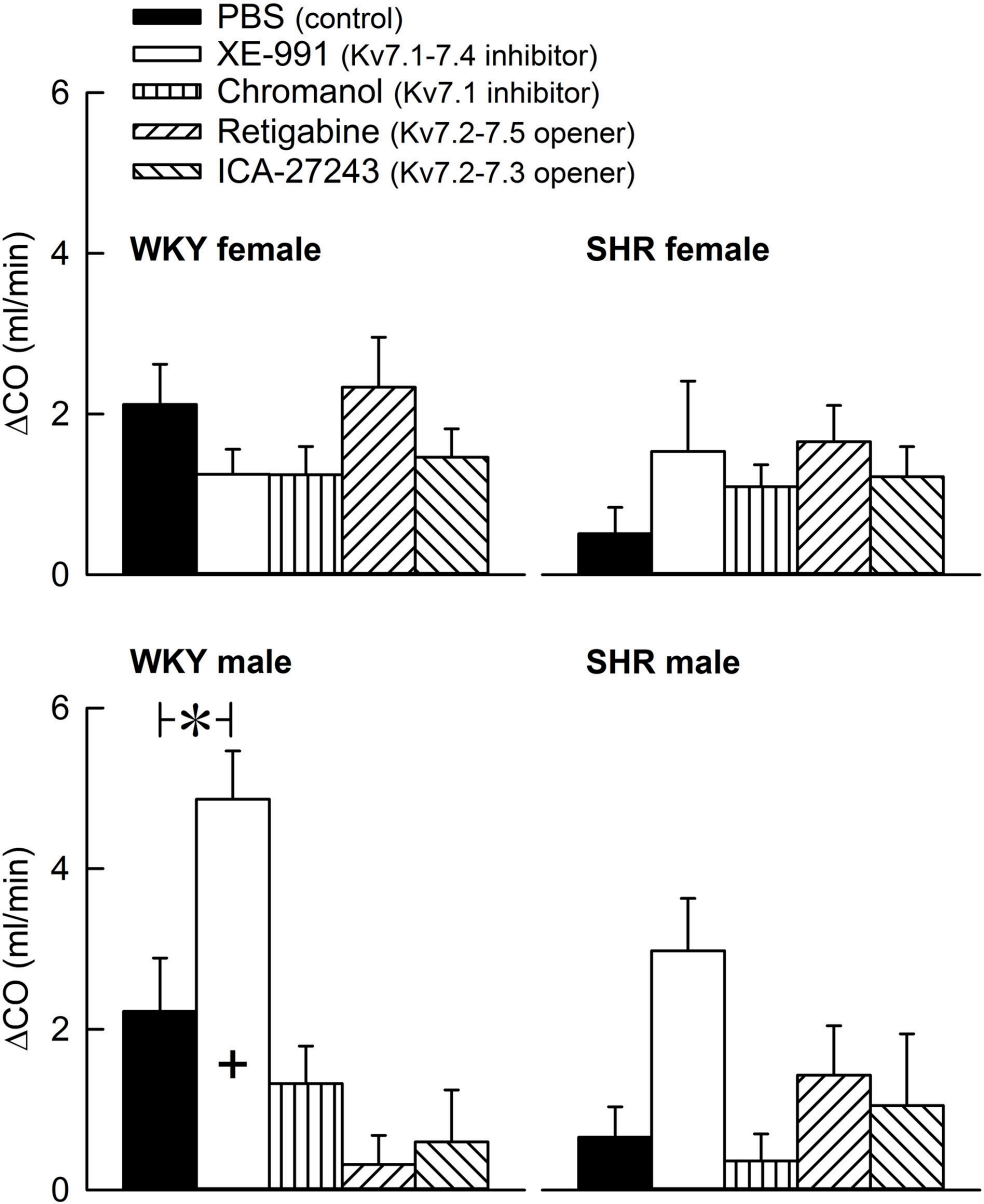
The acute effect of Kv7 inhibitors/openers on CO baseline. The rats were pre-treated with PBS (controls) or Kv7 inhibitor or opener, as indicated by the symbol legends. The change in CO compared to baseline was recorded at the time of the maximum change in TPR (Figure 2). Comparisons were made between corresponding PBS-control and experimental groups, and for corresponding experimental groups across sex (+ within column) or strain (none detected). Analyses were done by one-way ANOVA, followed by Student's *t*- or Kruskal–Wallis test. ^*^P ≤ 0.0125.

**Figure 5:**
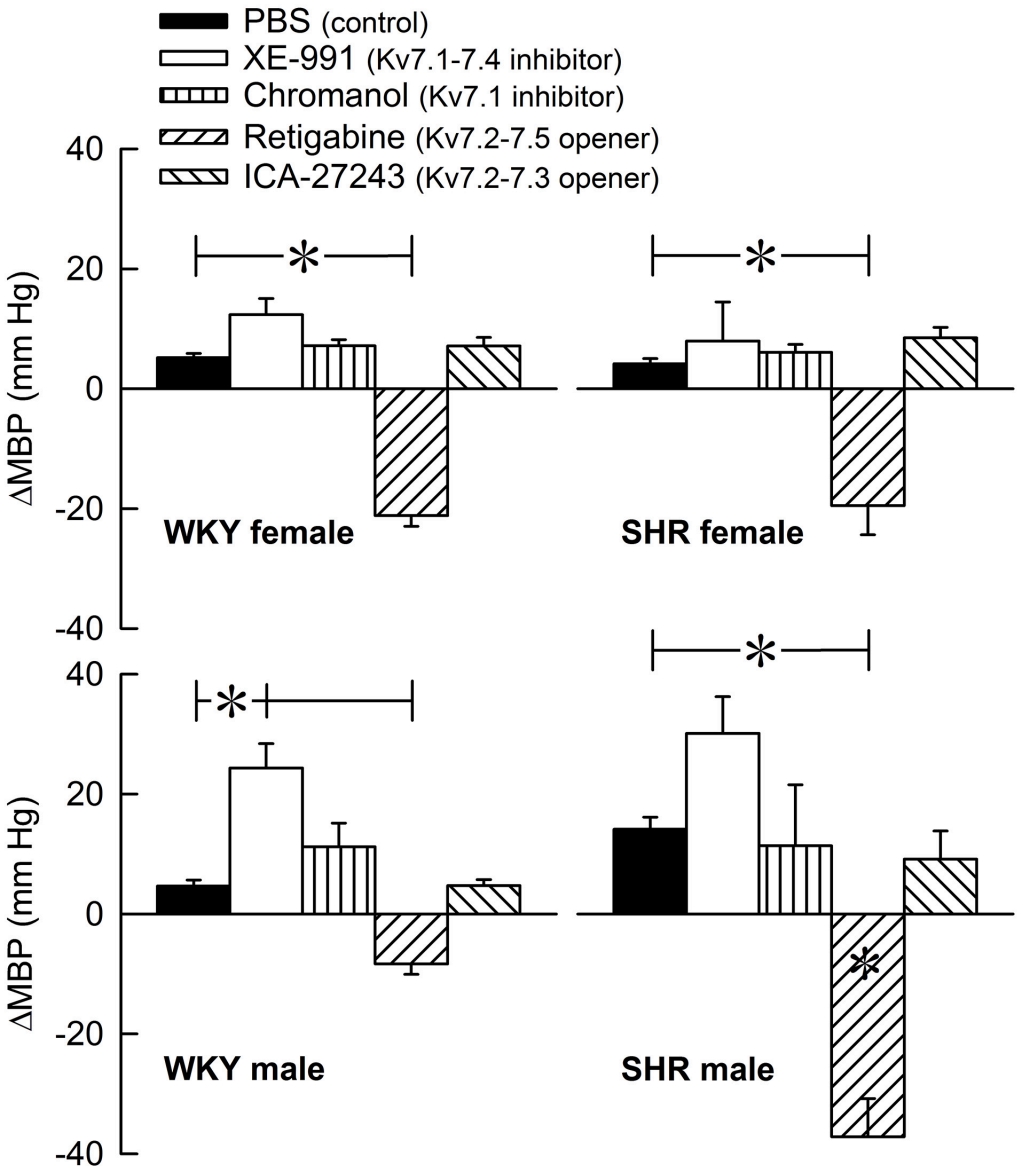
The acute MBP-response to Kv7 inhibitors/openers. The MBP-response was recorded at the time of maximum change in TPR (Figure 2). The rats were injected with PBS (controls) or Kv7 inhibitor or opener, as indicated by the symbol legends. Comparisons were made between corresponding PBS-control and experimental groups, and for corresponding experimental groups across sex (none detected) or strain (^*^within column). Analyses were done by one-way ANOVA, followed by Student's *t*- or Kruskal–Wallis test. ^*^P ≤ 0.0125.

The Kv7 inhibitor XE-991 induced a transient rise in baseline TPR in both sexes in WKY (*P* ≤ 0.002), reaching a maximum after about 6 min (Figure 2). In SHR, the response was not significantly different from that following the sham injection with PBS in the controls. The XE-991-induced bradycardia (Figure 3), recorded at the same time as the TPR-peak-response, was significant in the female WKY (*P* = 0.005) and male SHR (*P* ≤ 0.023). A rise in CO was detected only in the male WKY (*P* = 0.006) (Figure 4). These changes resulted in an XE-991-induced increase in baseline MBP in the male WKY only (*P* = 0.005) (Figure 5). Chromanol had no significant effect on the cardiovascular baselines (Figures 2–5). Myokymia in the facial, thoracic and abdominal muscles was seen in response to XE-991, but not chromanol. The Kv7 opener retigabine reduced baseline TPR, HR and MBP in all groups, reaching a minimum within 1 min (*P* ≤ 0.001) (Figures 2, 3, 5), but had no effect on baseline CO (Figure 4). ICA-27243 had no effect on the cardiovascular baselines (Figures 2–5). A sex-related difference was detected only in the CO-response to XE-991 (male>female in WKY, *P* = 0.001) (Figure 4), and a strain -related difference only in the TPR-, HR-, and MBP-response to retigabine (SHR>WKY in the males, *P* ≤ 0.012) (Figures 2, 3, 5). The response to channel inhibitors/openers was mostly transient, and at the time when the subsequent tyramine-infusion was started, no longer different from that prior to pre-treatment. However, a reduction in MBP of −17 ± 3 mm Hg was detected after retigabine in the female WKY and an increase in CO of 10 ± 2 ml/min after XE-991 in the male WKY (*P* ≤ 0.002).

### The effect of Kv7 channels on the cardiovascular response to tyramine

**Figure 6:**
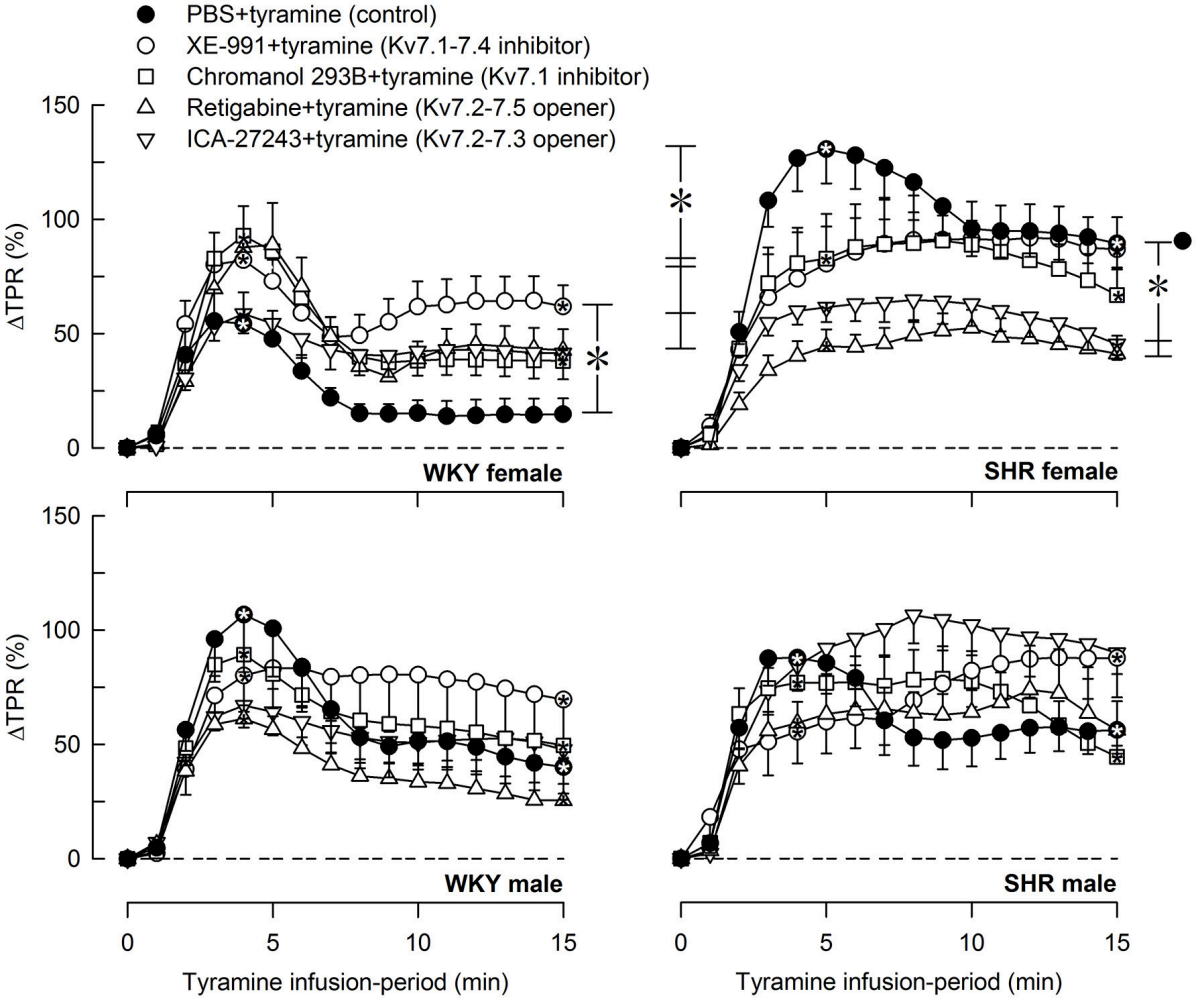
The effect of Kv7 inhibitors/openers on the TPR-response to tyramine-stimulated norepinephrine release. The rats were pre-treated as indicated by symbol legends. Analyses of the tyramine-response curves using Repeated Measures Analyses of Variance and Covariance indicated the presence of significant responses in all four sets of experiments, as well as the presence of differences between the control and the experimental groups within the female WKY and SHR rats, but not male rats. Significant responses, i.e., differences compared to base-line (^*^ within symbol), were subsequently located as indicated by one-sample Student's *t*-tests at the initial TPR-peak-response, i.e., at 4 min, and at the end of the tyramine-infusion, i.e., at 15 min. Significant differences between the control and the drug-treated, experimental groups within each set of experiments were located by two-sample Student's *t*- or Kruskal–Wallis tests at 4 min (^*^ within brackets left of curves) and at 15 min (^*^ within brackets right of curves) as indicated. In the order of symbol legends, TPR prior to tyramine = 3.0 ± 0.2, 2.4 ± 0.2, 2.5 ± 0.2, 2.5 ± 0.1, 2.1 ± 0.2 mm Hg/ml/min in the female WKY groups, 3.2 ± 0.3, 4.1 ± 0.5, 3.4 ± 0.4, 4.3 ± 0.4, 5.3 ± 0.4 mm Hg/ml/min in the female SHR groups, 2.1 ± 0.2†,1.7 ± 0.1, 2.8 ± 0.2^‡^, 1.9 ± 0.2, 2.7 ± 0.3 mm Hg/ml/min in the male WKY groups, and 4.4 ± 0.3^*^†, 3.2 ± 0.2^‡^, 5.2 ± 0.6, 3.5 ± 0.3, 4.5 ± 0.5 mm Hg/ml/min in the male SHR groups (*P* ≤ 0.0125 for strain- (^*^), sex- (), or drug- (^‡^) related differences in the baselines). ^*^ within symbol and brackets —*P* ≤ 0.025 (please see the Materials and Methods section for further details).

The tyramine-induced release of norepinephrine resulted in a rise in TPR, which returned to baseline in the female WKY, but remained elevated in the female SHR and in male rats of both strains (*P* = NS and ≤0.006 at 15 min, respectively) (Figure 6). The TPR-response in the female SHR was somewhat greater than that in the male SHR and much greater than that in the female WKY throughout the tyramine-infusion-period (*P* ≤ 0.004). The TPR-response in the female WKY was less than that in the male WKY (*P* ≤ 0.004). XE-991 enhanced the TPR-response during the late part of the tyramine-infusion period in the female WKY (*P* = 0.005). In contrast, in the female SHR, XE-991, chromanol, retigabine and ICA-27243 all reduced the initial TPR-peak-response (*P* ≤ 0.005), whereas only retigabine and ICA-27243 reduced the TPR-response during the late part of the tyramine-infusion period (*P* ≤ 0.001) (Figure 6).

**Figure 7:**
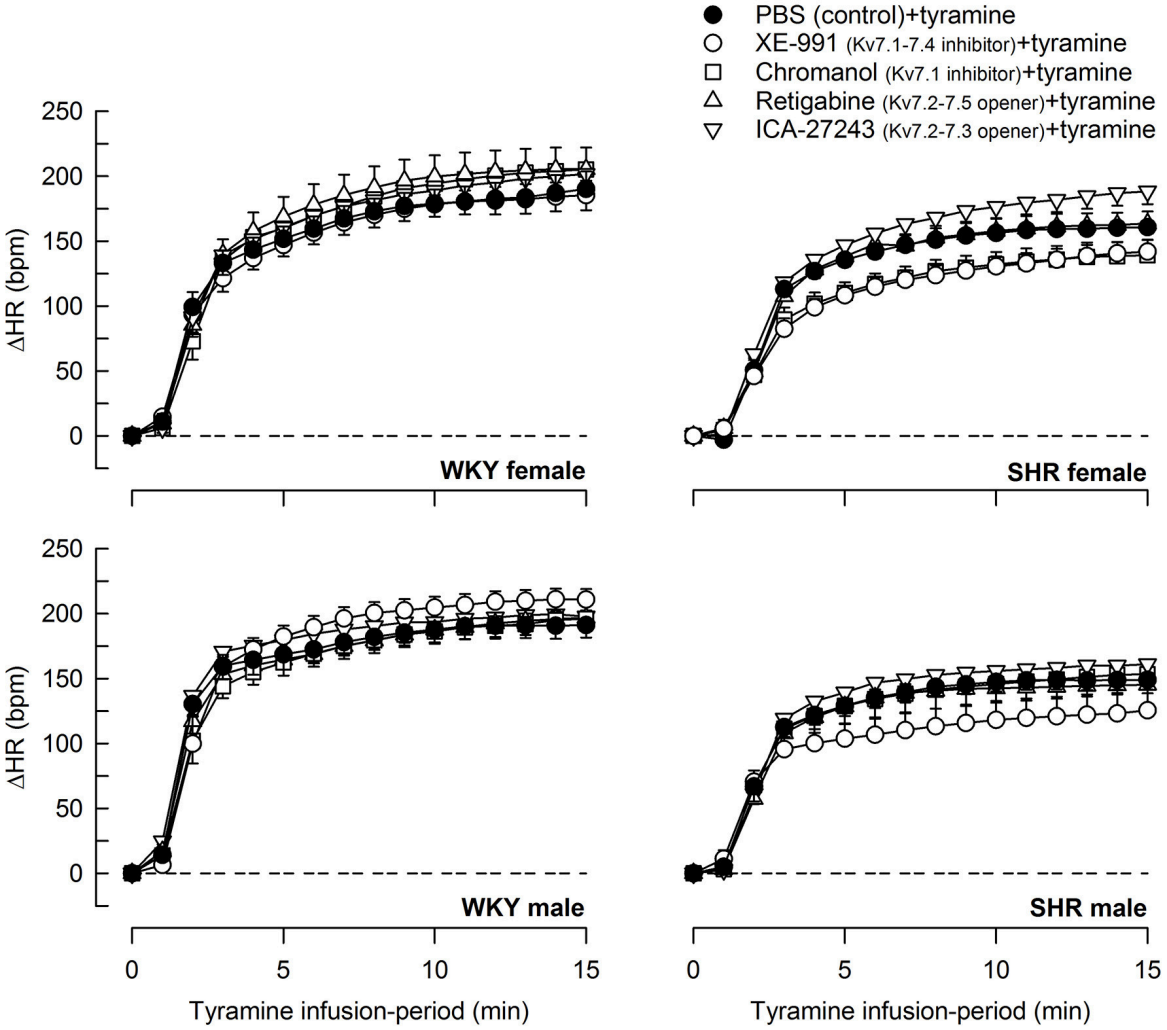
The effect of Kv7 inhibitors/openers on the HR-response to tyramine-stimulated norepinephrine release. The rats were pre-treated as indicated by symbol legends. In the order of symbol legends, HR prior to tyramine = 354 ± 12, 342 ± 6, 350 ± 9, 330 ± 19, 372 ± 11 bpm, 341 ± 8, 329 ± 7, 355 ± 7, 330 ± 9, 342 ± 14 bpm, 347 ± 11, 326 ± 8, 351 ± 7, 332 ± 8, 353 ± 6 bpm, and 379 ± 6†, 356 ± 9, 397 ± 17, 368 ± 9, 381 ± 11 bpm in female WKY and SHR and male WKY and SHR, respectively (†*P* ≤ 0.0125 for a sex-difference in the baselines in SHR. Strain- or drug-related differences were not detected). The HR-response curves to tyramine were analyzed using Repeated Measures Analyses of Variance and Covariance, followed by one- and two-sample Student's *t*-tests or Kruskal–Wallis tests (please see the Materials and Methods section for details). The HR-response to tyramine at 15 min was significantly different from baseline in all groups (*P* ≤ 0.001, not indicated). Significant differences between the control and the drug-treated experimental groups at 15 min were not detected.

**Figure 8:**
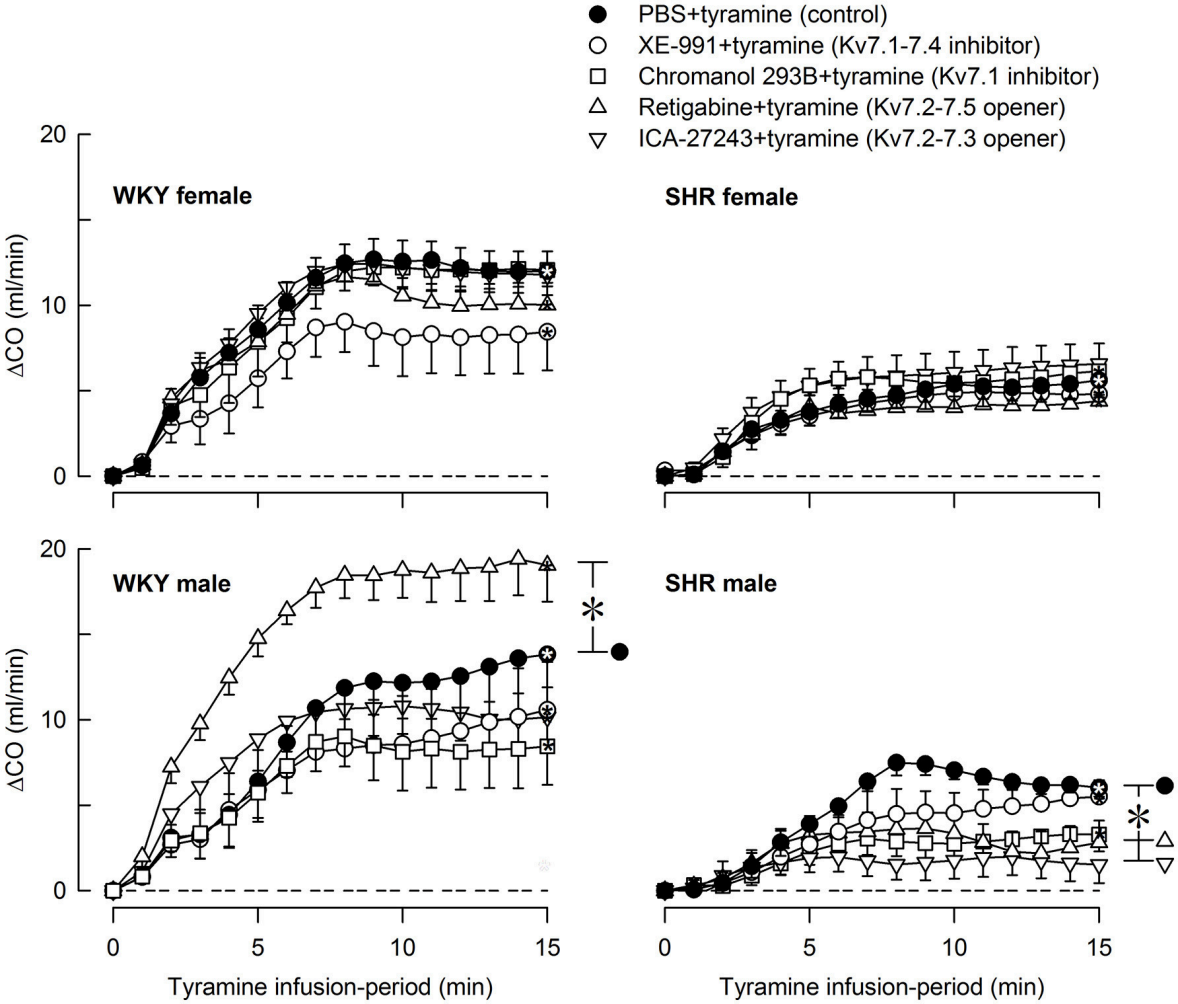
The effect of Kv7 inhibitors/openers on the CO-response to tyramine-stimulated norepinephrine release. The rats were pre-treated as indicated by legend symbols. Analyses using Repeated Measures Analyses of Variance and Covariance indicated the presence of significant responses in all four sets of experiments, as well as the presence of group differences within the male but not female WKY and SHR. Significant differences compared to baseline were detected at 15 min as indicated by the use of one-sample Student's *t*-tests (^*^within symbol). Differences between the control and the drug-treated, experimental groups within the four sets of experiments at 15 min were detected as indicated by two-sample Student's *t*-tests or Kruskal–Wallis tests (^*^ within brackets right of curves). The tyramine-induced rise in CO was clearly less in SHR than in WKY in both sexes (*P* ≤ 0.002), and was slightly less in the female than in the male in SHR (*P* = 0.011). A sex-dependent difference was not detected in WKY in spite of the difference in body weight. CO baselines prior to tyramine was 19 ± 1, 26 ± 2, 23 ± 1, 18 ± 1, 19 ± 1 ml/min, 15 ± 1^*^, 12 ± 1^‡^, 12 ± 1, 10 ± 1^‡^, 9 ± 1^‡^ ml/min, 29 ± 2†, 38 ± 2^‡^, 23 ± 2^‡^, 26 ± 2, 23 ± 2 ml/min, and 16 ± 1^*^, 19 ± 2, 14 ± 2, 16 ± 1, 15 ± 1 ml/min in female WKY and SHR and male WKY and SHR, respectively (*P* ≤ 0.0125 for strain- (^*^), sex- (†), or drug- (^‡^) related differences in the baselines). ^*^ within symbols and brackets—*P* ≤ 0.05 (please see the Materials and Methods section for further details).

**Figure 9:**
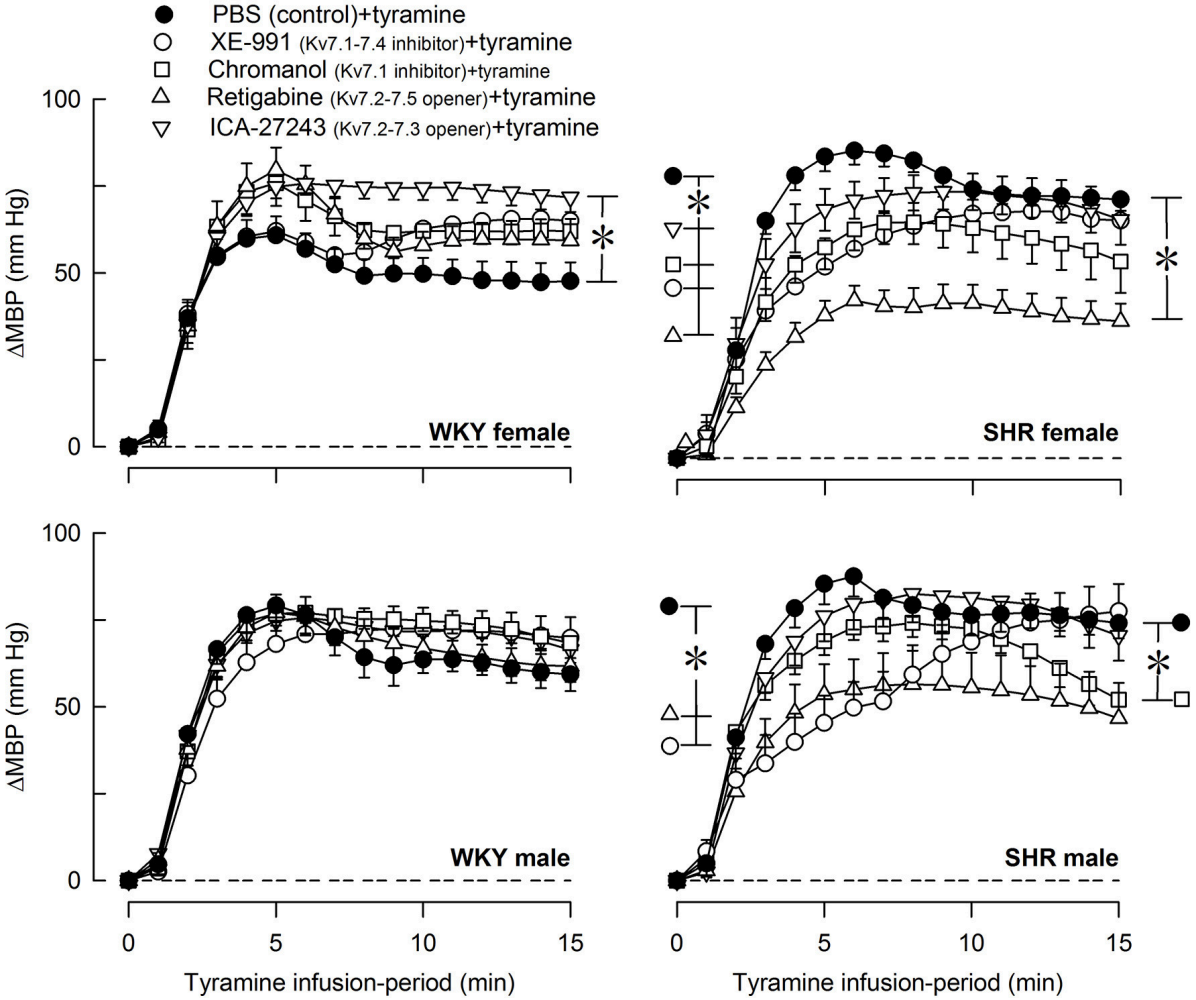
The effect of Kv7 inhibitors/openers on the MBP-response to tyramine-stimulated norepinephrine release. The rats were pre-treated as indicated by symbol legends. Differences between the control and the drug-treated experimental groups at the time of the initial TPR-peak-response, i.e., at 4 min (^*^within brackets left of curves), and at 15 min (^*^within brackets right of curves) were detected as indicated, using two-sample Student's *t*-tests or Kruskal–Wallis tests after curve evaluation with Repeated Measures Analyses of Variance and Covariance. The MBP-response at these times was significantly different from baseline in all groups (not indicated). In the order of symbol legends, MBP prior to tyramine = 56 ± 3, 61 ± 3, 56 ± 2, 44 ± 2^‡^, 59 ± 3 mm Hg, 57 ± 3, 50 ± 6, 42 ± 5, 42 ± 2, 49 ± 4 mm Hg, 57 ± 2, 63 ± 3^‡^, 62 ± 3, 51 ± 2, 58 ± 3 mm Hg, and 69 ± 4†, 59 ± 7, 69 ± 9, 55 ± 5, 67 ± 6 mm Hg in female WKY and SHR and male WKY and SHR sets of groups, respectively (*P* ≤ 0.0125 for strain- (^*^), sex- (), or drug- (^‡^) related differences in the baselines). ^*^P ≤ 0.025 (please see the Materials and Methods section for details).

Tyramine induced a sustained increase in HR (Figure 7), which was greater in WKY than in SHR in both sexes (*P* ≤ 0.003). The HR-response to tyramine was not influenced by the Kv7 inhibitors or openers. There was also a rise in CO, which was increased by retigabine in the male WKY, but reduced by retigabine and ICA-27243 in the male SHR (*P* ≤ 0.029) (Figure 8). The tyramine-induced changes in CO and TPR resulted in a rise in MBP (Figure 9), which was higher in SHR than in WKY in both sexes (*P* ≤ 0.024 at 15 min), whereas a sex-dependent difference was not detected. ICA-27243 increased the late MBP-response to tyramine in the female WKY (*P* = 0.009). XE-991, chromanol, retigabine and ICA-27243 reduced the MBP-response at 4 min (*P* ≤ 0.02) and retigabine also the response at 15 min (*P* < 0.001) in the female SHR (Figure 8). The MBP-response 4 min into the tyramine-infusion was lower after XE-991 and retigabine also in the male SHR (*P* ≤ 0.01), whereas a reduction at 15 min was seen after chromanol in the male SHR (*P* = 0.017).

## Discussion

The present study demonstrated a multifaceted role of Kv7 channels in BP homeostasis. The main findings were that the Kv7.1-7.4 inhibitor XE-991, but not the Kv7.1 inhibitor chromanol, enhanced neuronal and adrenal catecholamine release in both sexes in SHR, but not in WKY. XE-991, but not chromanol, enhanced the vasoconstrictory TPR- and MBP-response to tyramine-induced norepinephrine release in the female WKY, whereas the openers retigabine (Kv7.2-7.5) and ICA-27243 (Kv7.2-7.3) reduced the TPR-response throughout the tyramine-infusion in the female SHR. Kv7 did not influence the TPR-response to tyramine-stimulated norepinephrine release in male rats of either strain.

### Augmented Kv7 channel control of catecholamine release in SHR

**Figure 10:**
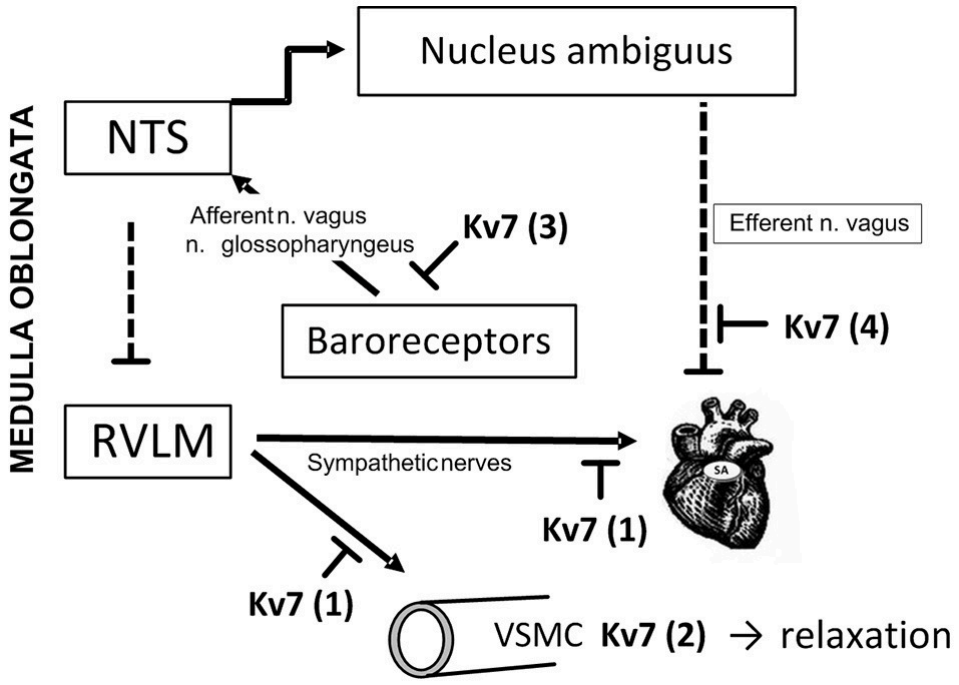
Kv7 channel targets in BP-homeostasis. Peripheral Kv7 channels may influence BP-control through several mechanisms. Through their hyperpolarizing effect, Kv7 channels may hamper transmitter release from sympathetic nerves (1), and through that reduce VSMC contraction and HR. This action may explain the enhanced tyramine-induced norepinephrine overflow (1) and epinephrine secretion from the adrenal glands (not indicated) in SHR of both sexes after pre-treatment with XE-991. A similar increase was not seen after chromanol, suggesting involvement of Kv7.2-7.3. In addition, Kv7 channels i VSMC may promote hyperpolarization, and thus oppose the vasoconstriction and rise in TPR following release of norepinephrine (2). This mechanism was likely to explain the augmented TPR-response to tyramine after XE-991 in female WKY. A similar increase was not seen after chromanol, suggesting involvement of VSMC Kv7.4. VSMC Kv7 channels were also likely to explain the reduced TPR-response to tyramine in female SHR pre-treated with the channel openers retigabine and ICA-27243. Channel openers/inhibitors did not alter the response to tyramine in male rats. VSMC Kv7 channels may therefore contribute to the delayed development of hypertension in women and female SHR. Due to the anesthesia in this experimental set-up, Kv7 channels located in baroreceptors and the nodose ganglion were not likely to influence the response to tyramine (3). Kv7 channels may hamper release of acetylcholine from parasympathetic neurons which inhibit catecholamine release. This mechanism may explain the increased catecholamine release after Kv7 openers in the female SHR (not indicated). Inhibition of vagal Kv7 channels (4) may explain the fall in resting HR in response to XE-991. NTS: nucleus tractus solitarii. RVLM: rostral ventrolateral medulla. Pointed arrows: Stimulating neural pathways. Blunted, dotted arrows: Inhibitory neuronal pathways. Blunted arrows: Inhibitory effects of Kv7.

Tyramine activated release of norepinephrine from sympathetic nerve endings (Figure 1), whereas the experiment itself activated some secretion of epinephrine from the adrenal glands. The present results indicated that Kv7.2-7.3-mediated hyperpolarization stabilized sympathetic neurons (Figure 10, option 1) as well as adrenal chromaffin cells in SHR, but not in WKY, and thus hampered release of both norepinephrine and epinephrine. This conclusion was based on the fact that the Kv7.1-7.4 inhibitor XE-991, but not the Kv7.1 inhibitor chromanol, increased the concentration of both catecholamines in plasma collected at the end of the experiment from SHR of both sexes, but not in plasma from WKY. A similar effect of XE-991 and chromanol on catecholamine release was seen in male SHR but not male WKY when the autonomic nervous system was activated by 3,4-diaminopyridine-induced depolarization (Berg, 2016b). Since Kv7.2-7.3 has been shown to be the predominant Kv7/M-channel in neurons (Wang et al., 1998), it seemed likely that the enhancing effect of XE-991 on catecholamine release in SHR reflected an up-regulation of Kv7.2-7.3 expression and/or function. The hyperadrenergic state in hypertension was therefore not explained by a reduced M-channel control of catecholamine release, on the contrary, up-regulation of Kv7.2-7.3 may represent a compensatory response in order to oppose an augmented catecholamine release in SHR.

Surprisingly, also pre-treatment with the Kv7.2-7.5 channel opener retigabine increased the plasma concentration of both catecholamines, but only in the female SHR. Also the Kv7.2-7.3 preferring opener ICA-27243 increased the plasma catecholamine concentrations, also in the female SHR alone. Since retigabine and ICA-27243 target channel opening at different sites of the Kv7 molecule (Padilla et al., 2009), it seemed reasonable to conclude that the effect of these two Kv7 openers on catecholamine release involved Kv7.2-7.3 rather than a non-Kv7 action. Furthermore, the increased plasma catecholamine concentrations after pre-treatment with Kv7 openers was statistically significant in the female SHR only, although the same tendency, but not statistically significant, was seen after retigabine in the male SHR. A similar effect was not seen at all in WKY of either sex. This strain-related selectivity was not likely to be seen if the effect of retigabine and ICA-27234 was due to a non-specific drug action. Opening of Kv7 channels in sympathetic neurons will lower catecholamine release, and an involvement of Kv7 channels in neurons which hamper catecholamine release may therefore be postulated. Kv7.2, 7.3, and 7.5 have been demonstrated in the afferent part of the baroreflex (Figure 10, option 3), in the nodose ganglion and in sensory baroreceptor terminals, where retigabine increased and XE-991 reduced the threshold for pressure-induced baroreceptor activation in an isolated aorta arch preparation (Wladyka and Kunze, 2006; Wladyka et al., 2008). But since the baroreflex is hampered by the anesthesia (Bjørnstad-Ostensen and Berg, 1994; Berg et al., 2012), Kv7 channels in the afferent limb were not likely to be involved. However, an up-regulation of Kv7 function in preganglionic neurons will lower acetylcholine release and activation of muscarinic receptors which inhibit Kv7 channels in the postganglionic sympathetic neuron. Hence, the name M-currents (Brown and Adams, 1980). Up-regulation of Kv7.2-7.3 in postganglionic parasympathetic neurons interacting with sympathetic neurons and adrenal chromaffin cells will have a similar effect on catecholamine release. The latter scenario was supported by studies showing that an augmented Kv7 function hampered vagal control of HR in the male SHR (Berg, 2016b) (Figure 10, option 4). Similar experiments have not been performed on the female SHR. Further studies are therefore required to establish the localization and the role of the retigabine- and ICA-27234-sensitive neurons in the female SHR.

### Kv7-influence on resting TPR

The increase in resting TPR in response to the Kv7.1-7.4 inhibitor XE-991 indicated that Kv7 channels represented a vasodilatory component in the control of resting TPR. The TPR-response to XE-991 was not likely to involve increased norepinephrine release, since it remained in male SHR pre-treated with reserpine, which eliminates norepinephrine from sympathetic nerve endings (Berg, 2016b). The Kv7 channels involved were therefore likely to be located in the VSMC. Since only Kv7.1, 7.4, and 7.5 have been detected in arterial VSMC (Greenwood and Ohya, 2009) and the Kv7.1 inhibitor had no effect on resting TPR, the XE-991-sensitive vasodilatory component was likely to be mediated by Kv7.4. However, the present result did not exclude an additional role of Kv7.5 in regulating VSMC tension. Clear strain- or sex-related differences were not observed, indicating that Kv7 currents, in the unstimulated condition, were not likely to contribute to the antihypertensive protection seen in the young female SHR.

The Kv7.2-7.5 opener retigabine, but not the Kv7.2-7.3 preferring opener ICA-27243, reduced resting TPR in all groups. The vasodilatory TPR-response to retigabine did not involve reductions in norepinephrine release, since it was observed also in reserpine-treated male SHR (Berg, 2016b). It therefore seemed that vasodilatory Kv7.4 channels, and possibly also Kv7.5, were present in all groups, most likely located in the VSMC. The open-state of these channels could be stimulated in all rats and then reduced resting TPR.

### Kv7-influence on resting HR

XE-991 precipitated a small reduction in HR baseline, although the difference was not statistically significant in all groups. Non-Kv7 effects of low doses of XE-991 have to my knowledge not been reported. The XE-991-induced bradycardia was therefore likely to result from a direct effect on efferent vagal nerves where inhibition of Kv7 channels may increase the release of acetylcholine (Figure 10, option 4). This conclusion was supported by the increased XE-991-induced bradycardia in male SHR after eliminating the counter-acting effect of norepinephrine by reserpine, and by that XE-991 increased the vagal influence on HR when both branches of the autonomic nervous system was activated by the non-Kv7 Kv inhibitor 3,4-diaminopyridine (Berg, 2016b).

A retigabine-induced bradycardia was seen in all groups and may result from a non-Kv7 action since it was present also in reserpinized male SHR (Berg, 2016b), thus excluding a role of stimulated Kv7 in sympathetic nerves. A non-Kv7 action was supported by the fact that the K_V_7.2-7.3-preferring channel opener ICA-27243 failed to lower resting HR. Indeed, retigabine has been shown to stimulate gamma-aminobutyric acid (GABA) synthesis and GABA-activated Cl^−^ channels (Kapetanovic et al., 1995; Rundfeldt and Netzer, 2000), which in the central nervous system may influence HR.

### Kv7-influence on the cardiovascular response to tyramine-stimulated norepinephrine release

M-channels are more likely to be open during depolarization (Brown and Passmore, 2009). Since the sympathetic tone is low in anesthetized rats, the effect of channel inhibitors/openers on the TPR-response to tyramine-stimulated norepinephrine release may better indicate how Kv7 modulate tension in VSMC in the awake condition, where VSMC are continuously stimulated by sympathetic nerves. Unlike that in the unstimulated condition, XE-991 increased the TPR-response to tyramine in the female WKY only. A parallel increase in catecholamine release was not detected, suggesting that vasodilatory VSMC Kv7 opposed the norepinephrine-induced vasoconstriction, but only in the female WKY. Since the Kv7.1 inhibitor chromanol did not increase the TPR-response to tyramine, the subtype involved was most likely Kv7.4. However, an additional role of Kv7.5, which is not inhibited by XE-991, could not be excluded. These results were compatible with the increased expression of Kv7.4 in mesenteric artery from female compared to male mice (Abbott and Jepps, 2016). The TPR-response to tyramine in the female WKY was not down-regulated by pre-treatment with the Kv7 openers retigabine or ICA-23243, suggesting that the VSMC Kv7.4 and 7.5 were fully open and could not be further stimulated.

In contrast, retigabine and ICA-27443 reduced the TPR-response throughout the tyramine-infusion period in the female SHR, suggesting that stimulation of hyperpolarizing VSMC Kv7 opposed the TPR-response to norepinephrine (Figure 1). This conclusion implied that ICA-27243, although Kv7.2-7.3-preferring, did not open Kv7.2-7.3 exclusively, and, indeed, ICA-27243 has been shown to influence also Kv7.4 (Wickenden et al., 2008). An involvement of VSMC rather than sympathetic nerve Kv7 channels was indicated since both retigabine and ICA-27243 increased overflow of norepinephrine in the female SHR. It was therefore concluded that VSMC Kv7.4 and possibly also Kv7.5 were present in the female SHR, but unlike that in the female WKY, vasodilatory Kv7.4-7.5 did not oppose the norepinephrine-induced rise in TPR unless stimulated by channel openers.

Although VSMC Kv7 channels in male rats of either strain responded to XE-991 (Berg, 2016b) and retigabine in the unstimulated condition, they did not oppose the vasoconstriction and did not respond to channel openers during tyramine-stimulated norepinephrine release. The reason for this was not disclosed by the present experiments. Kv7 channel expression and function is regulated by ancillary protein (β-) subunits and various intracellular signaling substances (Delmas and Brown, 2005; Fosmo and Skraastad, 2017). A sex-dependant difference in the ancillary protein KCNE4 has been detected by gene-deletion in the mouse, where KCNE4 enhanced Kv7 function primarily in the male (Abbott and Jepps, 2016). Clarifying if sex- and strain-dependant regulatory factors prevent Kv7 channels from counter-acting tyramine-induced vasoconstriction in the male may provide insight into how Kv7 channels may be manipulated to prevent development of hypertension.

The Kv7 inhibitors XE-991 and chromanol unexpectedly blunted the initial TPR-peak-response in the female SHR. The mechanism(s) underlying this observation was not clear, and further studies are required to fully understand this result.

The effect of Kv7 inhibitors or openers on the MBP-response to tyramine mostly paralleled the changes induced in TPR. The sustained tachycardia activated by the tyramine-stimulated norepinephrine release was not influenced by XE-991, chromanol, retigabine, or ICA-27243 in either strain or sex. The amount of tyramine-induced release of norepinephrine into the synapse was apparently greater than that needed for a maximum HR-response, and drug-induced differences in the plasma catecholamine levels therefore did not influence the HR-response.

In conclusion, this early attempt to disclose the complex role of Kv7 channels in cardiovascular control and hypertensive disease revealed some results which were readily explained, and others which will require further studies to fully understand the mechanisms involved. The data revealed an up-regulated Kv7-inhibition of stimulated catecholamine release in both sexes in SHR, most likely involving Kv7.2-7.3 in catecholamine-producing cells. Increased catecholamine release which was observed after channel openers in SHR, particularly in the female, may possibly involve Kv7.2-7.3 in cholinergic neurons with an inhibitory effect on catecholamine release. Both mechanisms will protect against excessive catecholamine release. Furthermore, VSMC Kv7.4 opposed norepinephrine-induced vasoconstriction in the female WKY only. However, in the female SHR, VSMC Kv7.4-7.5 could be stimulated by channel openers to counter-act adrenergic vasoconstriction. Kv7 inhibitors and openers did not influence the TPR-response to tyramine in male rats of either strain, possibly due to sex-dependent regulators of channel expression and/or function. Neuronal and VSMC Kv7 channels may therefore play important roles in BP homeostasis and represent future targets for antihypertensive therapy.

## Financial support

This work was supported by The Norwegian Council on Cardiovascular Diseases and Anders Jahre's Fond.

## Author contributions

The author confirms being the sole contributor of this work and approved it for publication.

### Conflict of interest statement

The author declares that the research was conducted in the absence of any commercial or financial relationships that could be construed as a potential conflict of interest.
